# Regulation of Interleukin-10 Receptor Ubiquitination and Stability by Beta-TrCP-Containing Ubiquitin E3 Ligase

**DOI:** 10.1371/journal.pone.0027464

**Published:** 2011-11-08

**Authors:** Hui Jiang, Yi Lu, Liang Yuan, Jianghuai Liu

**Affiliations:** 1 MOE Key Laboratory of Model Animals for Disease Study, Model Animal Research Center of Nanjing University and National Resource Center for Mutant Mice, Nanjing, China; 2 Zhejiang Key Lab for Technology & Application of Model Organisms, School of Life Science, Wenzhou Medical College, Wenzhou, China; Hungarian Academy of Sciences, Hungary

## Abstract

Interleukin-10 (IL-10) initiates potent anti-inflammatory effects via activating its cell surface receptor, composed of IL-10R1 and IL-10R2 subunits. The level of IL-10R1 is a major determinant of the cells' responsiveness to IL-10. Here, via a series of biochemical analyses using 293T cells reconstituted with IL-10R1, we identify the latter as a novel substrate of βTrCP-containing ubiquitin E3 ligase. Within the intracellular tail of IL-10R1, a canonical (^318^DpSGFGpS) and a slightly deviated (^369^DpSGICLQEP) βTrCP recognition motif can additively recruit βTrCP in a phosphorylation-dependent manner. βTrCP recruitment leads to ubiquitination, endocytosis and degradation of IL-10R1, subsequently reducing the cellular responsiveness to IL-10. Our study uncovers a novel negative regulatory mechanism that may potentially affect IL-10 function in target cells under physiological or pathological conditions.

## Introduction

Higher organisms have evolved sophisticated anti-inflammatory systems to prevent immuno-pathology. Among these systems, interleukin-10 (IL-10) exhibits a critical and indispensible role in limiting inflammation under numerous conditions (reviewed in [1]). Mechanistically, IL-10 is thought to mainly target innate immune cell types and antigen-presenting cells, partly via its powerful action in inhibiting pro-inflammatory cytokine synthesis. In addition, direct effects of IL-10 in suppressing T cells' function [2], [3], [4], or on the development of T regulatory lineages, have also been reported extensively [5], [6], [7]. Parallel to the diversity of IL-10 target cell types, IL-10 production itself can be attributed to immune cells of multiple lineages (reviewed in [8]). The wide distribution of IL-10 signaling circuit in the immune system confers multiple dimensions to the IL-10-associated immuno-regulation, which fittingly reflects the latter's role as a fundamental safeguard against the ‘unwanted’ aspects of immunity.

Based upon IL-10's potent immune-suppressive activity in cell culture and animal models, the potential of recombinant IL-10 in treating inflammatory diseases such as Crohn's disease and rheumatoid arthritis has long been considered. However, such a concept was only met with limited success in clinical trials (reviewed in [9]). The lack of significant therapeutic effects might result from the insufficient amount of recombinant IL-10 at the site of inflammation, as studies in animal models have shown the importance of a localized delivery of IL-10 in its efficacy of suppressing tissue-specific inflammation [10], [11]. In addition, since IL-10 also has some stimulating activities on the humoral [12], [13], [14] and cytotoxic [15], [16] arms of immunity, inability to control these confounding factors when systemically delivering IL-10 may have contributed to the dissatisfying results in such clinical trials. Therefore, the pharmacological manipulation of intracellular pathways that regulate IL-10 response appears to be a rational alternative to the usage of the recombinant ligand. Furthermore, it has been shown that in cells from arthritis joints or cells pre-treated with certain pro-inflammatory stimuli, IL-10 responses were indeed compromised [17], [18], [19], [20]. Such data suggest that a reduced action by IL-10 in target cells may be a critical pathogenic event underlying the development and/or progression of certain inflammatory conditions. In this regard, further identification of negative regulators of IL-10 signaling and exploration of their physiological/pathological roles will have significant therapeutic implications.

The molecular mechanisms whereby IL-10 mediates its biological function have been extensively studied. IL-10 activates its cell surface receptor complex, composed of IL-10R1 and IL-10R2 subunits. Both receptor subunits are critical for IL-10 functions [21], [22], [23]. The binding of IL-10 to its receptor results in activation of receptor-associated tyrosine kinases of Janus kinase family, Jak1 and Tyk2, which in turn lead to phosphorylation of two tyrosine residues (Y427, Y477 in mouse and Y446, Y496 in human) on IL-10R1. These phosphorylated tyrosine residues recruit Stat3 to the activated receptor, which causes the subsequent phosphorylation and activation of the latent transcription factor [24], [25]. There is strong evidence suggesting that Stat3, and particularly its extended duration of activation under IL-10 stimulation, are critical to the ligand's anti-inflammatory activity [26], [27], [28]. Therefore, regulatory mechanisms affecting the signaling events upstream of Stat3 can profoundly impact IL-10-mediated immune suppression.

IL-10 receptor complex represents the only ligand-specific signaling component within the proximal IL-10 pathway. Between the two receptor subunits, only IL-10R1 is exclusive for IL-10 signaling [29]. In contrast, IL-10R2 can form complexes with other cytokine receptor subunits and function also as part of the receptors for IL-22, IL-26 and IFNλ [29]. Intriguingly, cellular responsiveness to IL-10 has been positively correlated with the levels of IL-10R1 expression in different cell types [30], suggesting that the regulation of IL-10R1 expression levels might be a key mechanism modulating IL-10 function *in vivo*.

Earlier studies have demonstrated that the ligand-induced degradation of cell surface IL-10R1 functions to terminate IL-10 signaling [31], [32]. Although the detailed mechanisms underlying such regulation are yet to be established, these very studies have pointed to a role of protein stability in determining the levels of IL-10R1 expression and, subsequently, the magnitudes of cellular responses to IL-10. IL-10R1 belongs to the class II cytokine receptor family that also includes type I IFN receptor subunits (reviewed in [29]). Previous studies have comprehensively unveiled that the recruitment of SCF^βTrCP^ ubiquitin E3 ligase to type I IFN receptor serves as a convergence point of multiple signaling pathways that regulate the latter receptor's stability [33], [34], [35], [36], [37], [38], [39]. In the present study, we tested the possibility of IL-10R1 also being a target of βTrCP-mediated ubiquitination and down-regulation. Our data provide biochemical evidence supporting the involvement of such a novel regulatory mechanism in modulating the magnitudes of cellular responses to IL-10.

## Results

**Figure 1:**
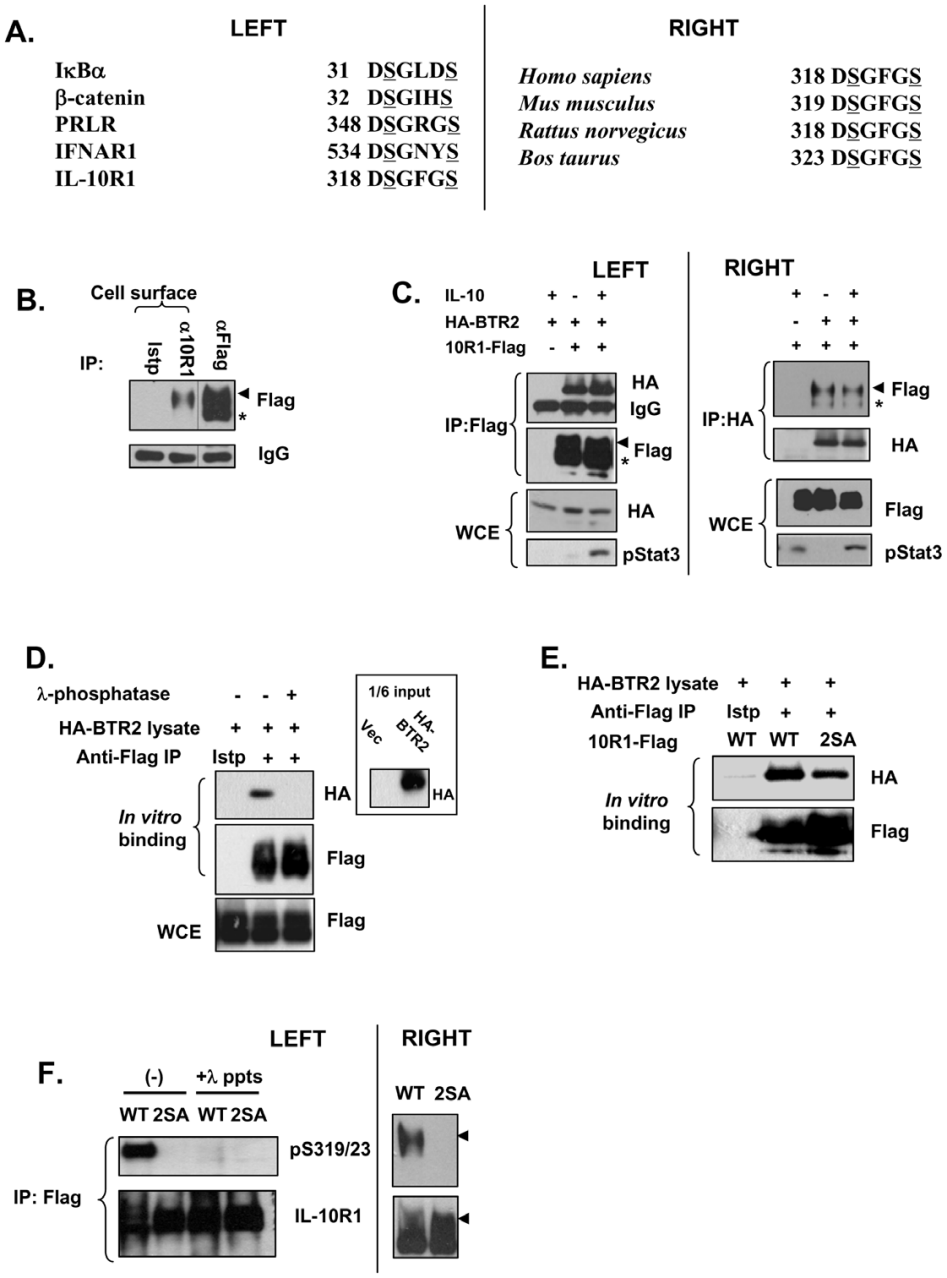
βTrCP can bind to the DSGFGS motif of IL-10R1. (A) Left: Alignment of some known βTrCP-binding sites together with the putative βTrCP site in human IL-10R1; right: putative βTrCP-binding sites in IL-10R1 from different species. Serine residues in IL-10R1 that might be equivalent to those in known βTrCP substrates, whose phosphorylation is required for βTrCP recognition, are underlined in a manner similar to those in the known substrates. (B) 293T cells transfected with IL-10R1-Flag were subjected to cell surface immunoprecipitation (IP) with the antibody against IL-10R1 (α10R1) or the isotype control IgG (Istp). Some cell lysates were subjected to regular IP with the antibody against Flag (αFlag). The IPed materials were analyzed on Western blot. The positions of the 120 kDa and the 100 kDa forms of IL-10R1 were marked by an arrow head and an asterisk respectively. (C) 293T cells were transfected with IL-10R1-Flag (10R1-Flag) and/or HA-βTrCP2 (HA-BTR2) and were treated with or without IL-10 (10 ng/ml) for 30 min before the lysates were harvested. The lysates were IPed using anti-Flag (left) or anti-HA (right). The IPed materials and the whole cell extracts (WCE) were analyzed on Western blot using indicated antibodies. The positions of the 120 kDa and the 100 kDa forms of IL-10R1 were marked as in (B). (D) Transfected IL-10R1 was purified from RIPA lysate of 293T cells via immunoprecipitation using Flag antibody. An isotype (Istp) control antibody IP was used as a negative control. The beads were treated with or without 200 U of λ-phosphatase for 30 min and then incubated at 4 degrees for 1 h with 50 µl of 293T lysates from cells transfected with HA-BTR2. The bound materials were analyzed on Western blot using indicated antibodies. The input levels of HA-BTR2 with the vector-transfected (Vec) sample as a negative control were shown in the inset. (E) Transfected WT or the S319, 23A (2SA) IL-10R1 was immunopurified from RIPA lysates and then subjected to *in vitro* binding assay using lysates from HA-BTR2-transfected cells. (F) WT or 2SA IL-10R1 was IPed from lysates of transfected 293T cells. Left: Some IPed materials were subjected to λ-phosphatase (λ-ppts) treatment or left untreated. The levels of Ser319, 23 phosphorylation and of the total IL-10R1 were determined. Right: Results presented are from a separate blot, where the 120 kDa and the 100 kDa forms of IL-10R1 are clearly discernible. The 120 kDa forms of phosphorylated and total IL-10R1 are marked by arrow heads.

We initially sought to use database search to identify candidate proteins that may represent novel βTrCP substrates. Many established SCF^βTrCP^ substrates (Fig. 1A, left) possess the DSG(X)_2+n_S consensus motifs (referred to as DSG motifs hereafter), where the two serine residues serve as βTrCP recognition sites when phosphorylated [40]. We used the above consensus sequence in our search of the Scansite database (http://scansite.mit.edu/ [41]). We noticed that human interleukin 10 receptor subunit 1 (IL-10R1) possesses such a motif (^318^DSGFGS^323^), which is conserved among various mammalian species (Fig. 1A, right). Since IL-10R1 belongs to the class II cytokine receptor family [29], which also includes type I interferon receptor subunit 1 (IFNAR1), an established substrate of SCF^βTrCP^ [33], we hypothesized that IL-10R1 may be subjected to a similar mode of regulation.

To examine the potential regulation of IL-10R1 by βTrCP, we utilized human embryonic kidney 293T cells as our major experimental system. These cells are devoid of functional endogenous IL-10R1 [42]. Transfected IL-10R1 appears on the Western blot as a 120 kDa form and a 100 kDa form (the arrow head and the asterisk in Fig. 1B), likely to be caused by different levels of glycosylation [43]. Via cell surface IP experiment, we found that the cell surface IL-10R1 runs at 120 kDa (Fig. 1B). We therefore will refer to the 100 kDa and the 120 kDa form as the immature (im) and the mature (m) IL-10R1 respectively. Since βTrCP interacts with its substrates to trigger their ubiquitination [40], we examined whether we could detect interaction between IL-10R1 and βTrCP, when the two are co-transfected in 293T cells. In such a system, HA-βTrCP2 could clearly co-immunoprecipitate (co-IP) with the C'-terminally Flag-tagged IL-10R1 (Fig. 1C, left), and a similar result was found with the reverse experiment (Fig. 1C, right). Notably, the mature form (120 kDa) of IL-10R1 was found to be associated with HA-βTrCP2 to a greater extent than the immature form (Fig. 1C, right: the arrow head and the asterisk), suggesting that βTrCP may preferentially target the mature IL-10R1. Interestingly, treatment of the cells with IL-10 did not considerably affect the levels of co-immunoprecipitated HA-βTrCP2 and IL-10R1-Flag (compare lane 2 and 3 in both left and right panels of Fig. 1C). Therefore, distinct from an established model applicable to several other cytokine receptors where ligand stimulation promotes βTrCP recruitment to the receptor [33], [44], [45], such an effect by IL-10 was not observed on ectopically over-expressed IL-10R1 used in the current study.

To further confirm that the ligand-independent interaction between IL-10R1 and βTrCP is specific, we first immuno-purified IL-10R1-Flag from transfected 293T cells in stringent RIPA buffer that significantly reduces the levels of indirectly pulled-down proteins [46]. Next, *in vitro* binding assay was performed by incubating the IL-10R1-bound beads with lysates from cells transfected with HA-βTrCP2 (Fig. 1D). Since the interaction of βTrCP with its substrate is mediated by phosphorylation of the substrate's DSG(X)_2+n_S motif, the immuno-purified IL-10R1 was also treated with λ-phosphatase. As shown in Fig. 1D, the binding of recombinant HA-βTrCP2 to IL-10R1 was critically dependent on IL-10R1 phosphorylation, as such was not observed using λ-phosphatase-treated receptor. Such a result also suggests that transfection of IL-10R1 is sufficient to cause its phosphorylation at the βTrCP recognition motif(s), in the absence of ligand treatment or other exogenous stimuli, a scenario similar to that previously observed with IFNAR1 [35]. We next examined whether the putative βTrCP-binding site (Ser319/23 in human IL-10R1 or Ser320/24 in mouse IL-10R1) mediates the receptor's interaction with βTrCP. To this end, we performed an *in vitro* binding assay using either the immuno-purified wild type (WT) IL-10R1 or its Ser319/23-to-Ala mutant (2SA). The substitution of these two conserved serine residues substantially reduced the binding of recombinant βTrCP2 to IL-10R1 (Fig. 1E), suggesting that the evolutionarily conserved DSGFGS motif is indeed a βTrCP recognition site. To directly examine the phosphorylation of Ser319/23 in IL-10R1, we generated a polyclonal antibody specific to phosphor-Ser319/23 (or Ser320/24 in mIL-10R1). Transfected WT IL-10R1, but not the 2SA mutant or the phosphatase-treated WT IL-10R1, could clearly be recognized by this antibody, demonstrating that Ser319 and Ser323 are phosphorylated in ectopically expressed IL-10R1 without ligand stimulation (Fig. 1F). A similar result was found with murine IL-10R1 (Figure S1). Additionally, in blots where the mature and immature forms of IL-10R1 were well separated, we clearly found that the Ser319, 23 phosphorylation is only associated with the mature form, but not the immature form (Fig. 1F, right). Such selective Ser319, 23 phosphorylation of the mature form of IL-10R1 may have contributed to the preferred binding of βTrCP to the latter, as seen in Fig. 1C. In sum, the data in Fig. 1 suggest that βTrCP directly interacts with mature IL-10R1 when the latter is phosphorylated at Ser319 and Ser323.

**Figure 2:**
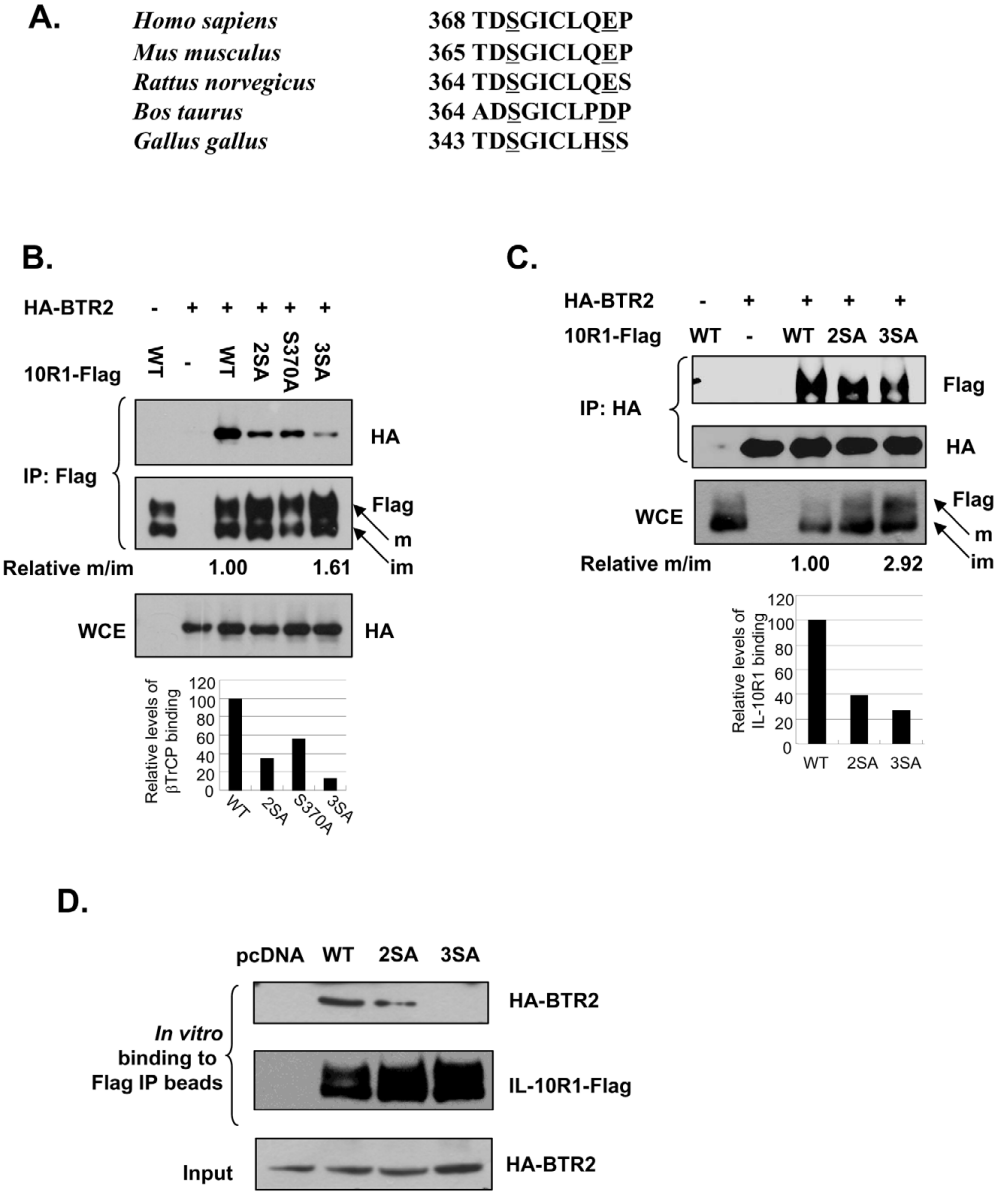
Identification of an additional βTrCP recognition motif in IL-10R1. (A) Alignment of the second putative βTrCP-binding site in IL-10R1 from different species. The conserved first serine residue and a second downstream negatively charged residue (or a serine) in such DSG motifs are underlined. (B) 293T cells were transfected with HA-BTR2 alone, or together with WT, S319, 23A (2SA), S370A or S319, 23, 70A (3SA) IL-10R1. Lysates were IPed with the Flag antibody. The IP or WCE samples were subjected to Western blot analysis. The relative levels of βTrCP binding to IL-10R1 were calculated according to the intensities of HA-βTrCP2 signal calibrated by the Flag-IL-10R1 signal. These values were presented by a bar graph underneath the HA panel of the WCE. Additionally, the relative ratio of abundance between the mature (m) and immature (im) IL-10R1 (pointed respectively by arrows) was determined for the WT or 3SA IL-10R1, and presented under the Flag blot (‘Relative m/im’, with the value of the WT set to 1.00). (C) Lysates from 293T cells transfected with the WT, 2SA or the 3SA IL-10R1, together with or without HA-BTR2 were IPed using HA antibody. The levels of bound and total IL-10R1, and levels of the HA-BTR2 were determined by Western blot analysis. The relative levels of IL-10R1 binding to HA-BTR2 were calculated according to the intensities of bound Flag-IL-10R1 signal calibrated by the total Flag-IL-10R1 signal. These values were presented by a bar graph below the picture panels. The relative ratio of abundance between the mature and immature WT or 3SA IL-10R1 (‘Relative m/im’, with the value of the WT set as 1.00) is presented under the Flag blot of the WCE samples. (D) 293T cells transfected with the WT, 2SA or the 3SA IL-10R1 were IPed using Flag antibody in RIPA buffer. The immunoprecipitates were washed extensively and then incubated with the cell lysates (50 µl) containing HA-BTR2. The levels of the bound HA-BTR2 as well as those of the IPed IL-10R1 were analyzed by Western blot. The levels of input HA-BTR2 (5 µl) were also presented.

It is noteworthy to point out that although phosphorylated Ser319 and Ser323 are likely to play a major role in the recruitment of βTrCP, the Ser319/23A IL-10R1 immuno-purified from the cells was not completely deficient in βTrCP binding *in vitro* (lane 3 of Fig. 1E). These results strongly suggest that the transfected IL-10R1 may possess additional phosphorylation-dependent βTrCP- binding site(s). In further surveying the sequences of IL-10R1 from different species, we identified another putative βTrCP-binding site (^369^DSGICLQEP) that is conserved in higher organisms (Fig. 2A). Interestingly, the Glu376 in human IL-10R1 is represented by Asp372 and by Ser351 in its bovine and chicken counterparts, respectively. We hypothesized that the negative charge on the Glu376 residue (or its equivalents in IL-10R1 from other mammalian species) may have functionally substituted for a phosphorylated second serine in the canonical DSG motif, and that βTrCP recruitment via this more membrane-distal site in mammalian IL-10R1 is triggered by phosphorylation of Ser370 (or its equivalents). To examine whether such a putative DSG motif in transfected IL-10R1 contributes to βTrCP binding in addition to Ser319/23, we generated additional IL-10R1 mutants harboring Ala substitutions at Ser370 alone (Ser370A), or at Ser319, Ser323 and Ser370 all together (3SA). Immuno-precipitation (IP) of IL-10R1 mutated at either the upstream (2SA) or the downstream (S370A) DSG motif yielded reduced levels of bound HA-βTrCP2 (65% and 43% reduction respectively), compared to that from the IP of WT IL-10R1 (Fig. 2B). Importantly, IL-10R1 mutated at both DSG motifs (3SA) exhibited an even more severe deficiency to co-immunoprecipitate HA-βTrCP2 (87% reduction, Fig. 2B). These data suggest that the two DSG motifs in transfected IL-10R1 act in an additive manner to mediate βTrCP-binding. The latter notion was further confirmed by the reverse co-IP experiment based on immuno-precipitation of HA-βTrCP2, which showed that compared to that of the WT IL-10R1, the efficiency of the 2SA and 3SA IL-10R1 to be pulled down together with HA-βTrCP2 reduced 61% and 74%, respectively (Fig. 2C). Moreover, a reciprocal IP experiment using the DSG motif mutants (Ser320/24A and Ser320/24/67A) of mouse IL-10R1 and HA-βTrCP2 has corroborated the findings with the human IL-10R1 (Figure S2). Lastly, an *in vitro* binding assay using stringently immuno-purified IL-10R1 has shown that the deficient recruitment of βTrCP by 3SA IL-10R1 was more severe than that by its 2SA counterpart (Fig. 2D), which serves as an additional proof for the presence of two functional βTrCP-binding sites in IL-10R1.

Therefore, the 3SA IL-10R1 represents a more severely affected βTrCP binding-deficient mutant form. It is interesting to note that the relative levels of the 3SA IL-10R1 were consistently higher than those of the WT IL-10R1 in these transient transfection experiments (Fig. 2B, 2C: Flag immunoblot panels). Moreover, compared to that of the WT IL-10R1, the mature form of the 3SA IL-10R1 makes a greater contribution to the abundance of the total (mature and immature) receptor (Fig. 2B, 2C: ‘m’- and ‘im’-denoted arrows and the ‘Relative m/im’ values). Importantly, similar results were obtained when the WT and 3SA IL-10R1 levels were measured using another antibody recognizing the N-terminus of the protein (Figure S3). Taken together with the data that βTrCP preferentially binds the mature IL-10R1 (Fig. 1C, right), these observations suggest that the additive interaction with βTrCP via both DSG motifs under the context of mature IL-10R1 results in the latter’s down-regulation.

**Figure 3:**
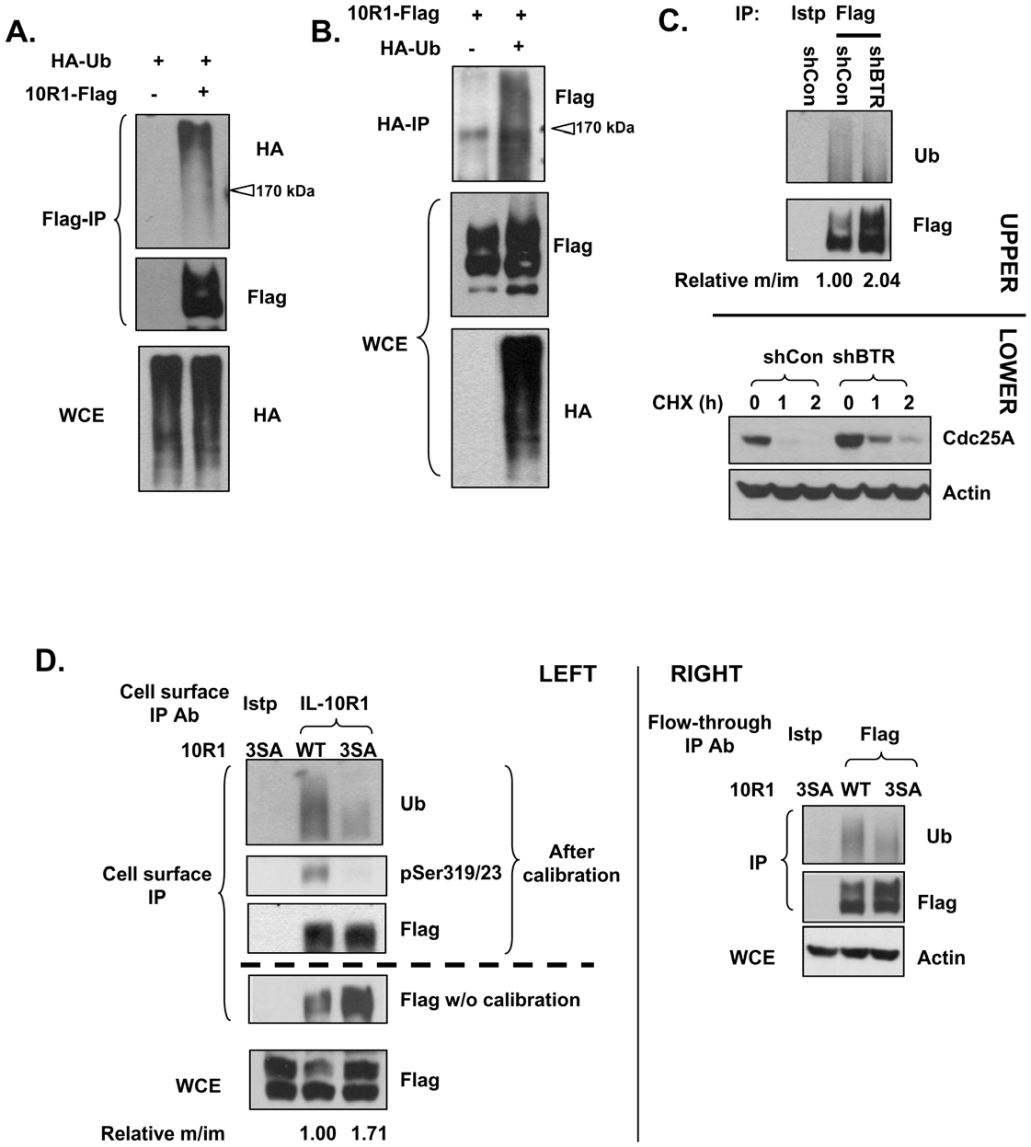
βTrCP mediates IL-10R1 ubiquitination. (A and B) 293T cells were transfected with IL-10R1-Flag and/or HA-ubiquitin (HA-Ub). Samples were subjected to denaturing IP with Flag (A) or HA (B) antibodies. The IP samples were analyzed for IL-10R1 ubiquitination by Western blot using HA (A) or Flag (B) antibodies. The positions of the 170 kDa on the immunoblots analyzing ubiquitination were marked by open arrow heads. (C) 293T cells were transfected with IL-10R1 and shRNA control (shCon) or shRNA against βTrCP1/2 (shBTR). Cellular proteins were subjected to denaturing IP using Flag antibody and the levels of ubiquitination were analyzed (upper panels). The relative ratio of abundance between the mature and immature IL-10R1 (‘Relative m/im’, with the value of the control sample set as 1.00) is presented under the Flag blot. In the lower panels, the degradation kinetics of Cdc25A (another βTrCP substrate) were compared between experiment groups using cycloheximide (CHX) to show the effectiveness of βTrCP knockdown. (D) 293T cells were transfected with the WT or 3SA IL-10R1. Cells were subjected to cell surface IP first (left panels), and the remaining fractions were subjected to another round of IP (‘Flow-through IP’, right panels). All natively IPed materials were denatured and then IPed with the antibody against Flag. For the cell surface IP samples (left panels), the un-calibrated levels of IL-10R1 were determined (‘Flag w/o calibration’). Next, the adjusted amounts of WT or the 3SA samples were loaded, so that each contains an equivalent level of IL-10R1 (the ‘After calibration’ bracket). The levels of ubiquitinated cell surface IL-10R1 were analyzed in the calibrated samples. As a control, the IL-10R1 levels within the WCE samples were shown in the lowest panel. The relative ratios of abundance between the mature and immature forms (‘Relative m/im’, with the value of the WT set as 1.00) of the WT or the 3SA receptor are presented underneath. In a parallel analysis (right panels), the levels of IL-10R1 ubiquitination were examined from the remaining cellular fractions after the cell surface IP.

The interaction between βTrCP and IL-10R1 may result in SCF^βTrCP^-dependent IL-10R1 ubiquitination. We used HA-tagged ubiquitin to examine whether co-transfected IL-10R1 is constitutively ubiquitinated in cells. A smeared, high-molecular-weight HA immuno-reactivity characteristic of protein poly-ubiquitination was observed in denaturing Flag-IL-10R1 IP (Fig. 3A). Importantly, a smear of Flag immuno-reactivity from the denaturing IP of HA-ubiquitin was detected in a parallel experiment, strongly suggesting the direct ubiquitination of IL-10R1 protein (Fig. 3B). To functionally establish that βTrCP is responsible for the constitutive ubiquitination of transfected IL-10R1, we performed βTrCP knockdown experiments. To avoid artifacts that may be caused by transfection of exogenous ubiquitin, we focused on examining IL-10R1 modification by the endogenous ubiquitin. Since Cdc25A is an established target of SCF^βTrCP^ [47], [48], the efficiency of βTrCP knockdown was shown by the increased stability of Cdc25A (Fig. 3C, lower panels). As anticipated, βTrCP knockdown markedly reduced the levels of constitutive IL-10R1 ubiquitination (Fig. 3C, upper panels). It is important to note that similar to mutating the βTrCP-binding sites of IL-10R1 (Fig. 2B, 2C), βTrCP knockdown also led to an increase in the relative levels of the mature WT IL-10R1, in comparison to its immature counterpart (Fig. 3C, ‘Flag’ panel and the values of ‘Relative m/im’). Since ubiquitination of a given protein often leads to its selected down-regulation, these data further imply that between the mature and immature forms of IL-10R1, the former is a preferred target of βTrCP-mediated ubiquitination.

To examine the cellular location where βTrCP-mediated constitutive IL-10R1 ubiquitination occurs, we sequentially enriched the cell surface IL-10R1 (antibody-labeling of live cells) and its counterpart within the remaining cellular fraction. Cell surface levels of the βTrCP binding-deficient, 3SA IL-10R1, are significantly higher than those of WT IL-10R1 after transient transfection (Fig. 3D left, the panel of ‘Flag w/o calibration’), consistent with the patterns of the mature IL-10R1 in the whole cell lysates (Fig. 3D left and Fig. 2B, 2C). After calibration of IL-10R1 levels, the ubiqitination of cell surface 3SA IL-10R1 was found to be substantially diminished, compared to that of the cell surface WT IL-10R1 (Fig. 3D left: the ‘Ub’ panel). These results indicate that βTrCP binding contributes to the direct ubiquitination and down-regulation of the cell surface IL-10R1. Interestingly, in the fractions that had the initial antibody-bound IL-10R1 removed (‘Flow-through IP’), the levels of ubiquitinated WT and 3SA IL-10R1 followed a similar pattern (Fig. 3D, right). Although not exclusively, IL-10R1 within the latter fraction mainly comes from the intracellular pool. Therefore, these very results imply that the mature IL-10R1 within the intracellular pool may also undergo βTrCP-dependent ubiquitination. Nevertheless, it remains possible that a certain portion of the ubiquitinated, intracellular IL-10R1 results from the endocytic trafficking of the ubiquitinated cell surface IL-10R1.

**Figure 4:**
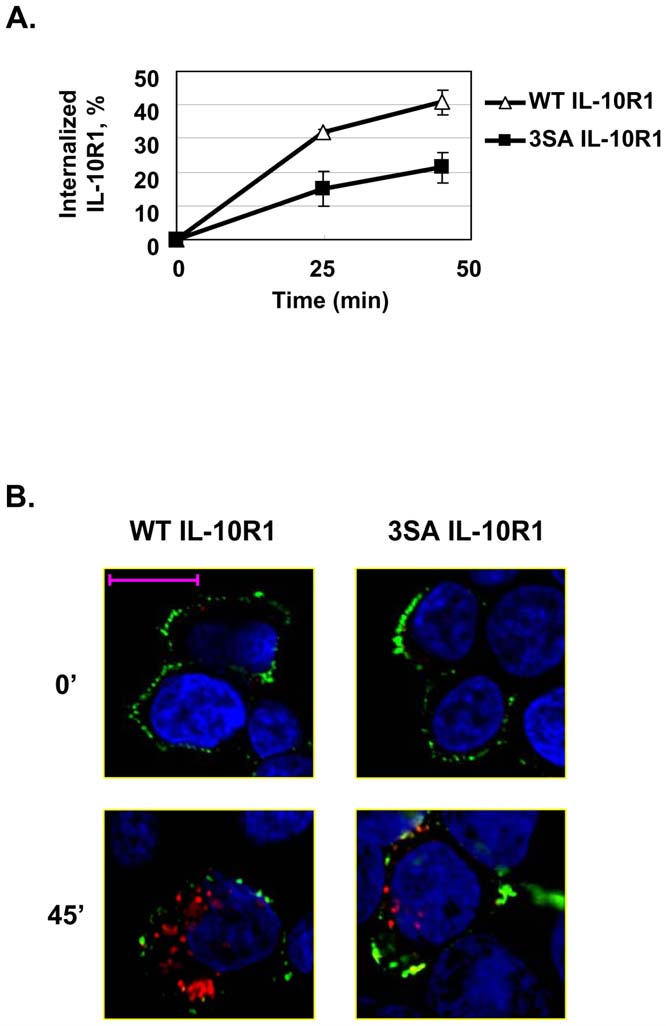
βTrCP-mediated ubiquitination leads to endocytosis of IL-10R1. (A) 293T cells stably transfected with the WT IL-10R1 (open triangles) or the 3SA (black squares) IL-10R1 were subjected to an ELISA-based endocytosis assay. Cells were allowed to endocytose antibody-labeled IL-10R1 for the indicated periods of time. The cells were then incubated with a HRP-conjugated secondary antibody. The amounts of labeled IL-10R1 remaining on the cell surface were determined by measuring the cell-associated HRP activity. Two wells were used for every experimental condition and the average values are presented on the graph. The error bars depict the range of data from the duplicated wells. A representative result from one of two independent experiments is presented. (B) The same cells as in (A) were pre-labeled with IL-10R1 antibody. After being allowed to endocytose the antibody-labeled IL-10R1 for 45 min, the cells were fixed with paraformaldehyde. The fixed cells were incubated with an Alexa488-conjugated secondary antibody to stain the remaining cell surface IL-10R1. Next, the same samples were permeablized and incubated with a second Cy3-conjugated anti-rat IgG to stain the endocytosed IL-10R1. The nuclei were labeled with DAPI. The representative three-color-overlaid pictures of cells expressing WT or 3SA IL-10R1 before (0') and after (45') initiation of endocytosis are presented (scale bar = 10 µm). Since our stable cell line selection scheme was based on the drug resistance of a co-transfected plasmid, not all cells expressed IL-10R1. The negatively stained cells serve as internal controls for the antibody specificity.

**Figure 5:**
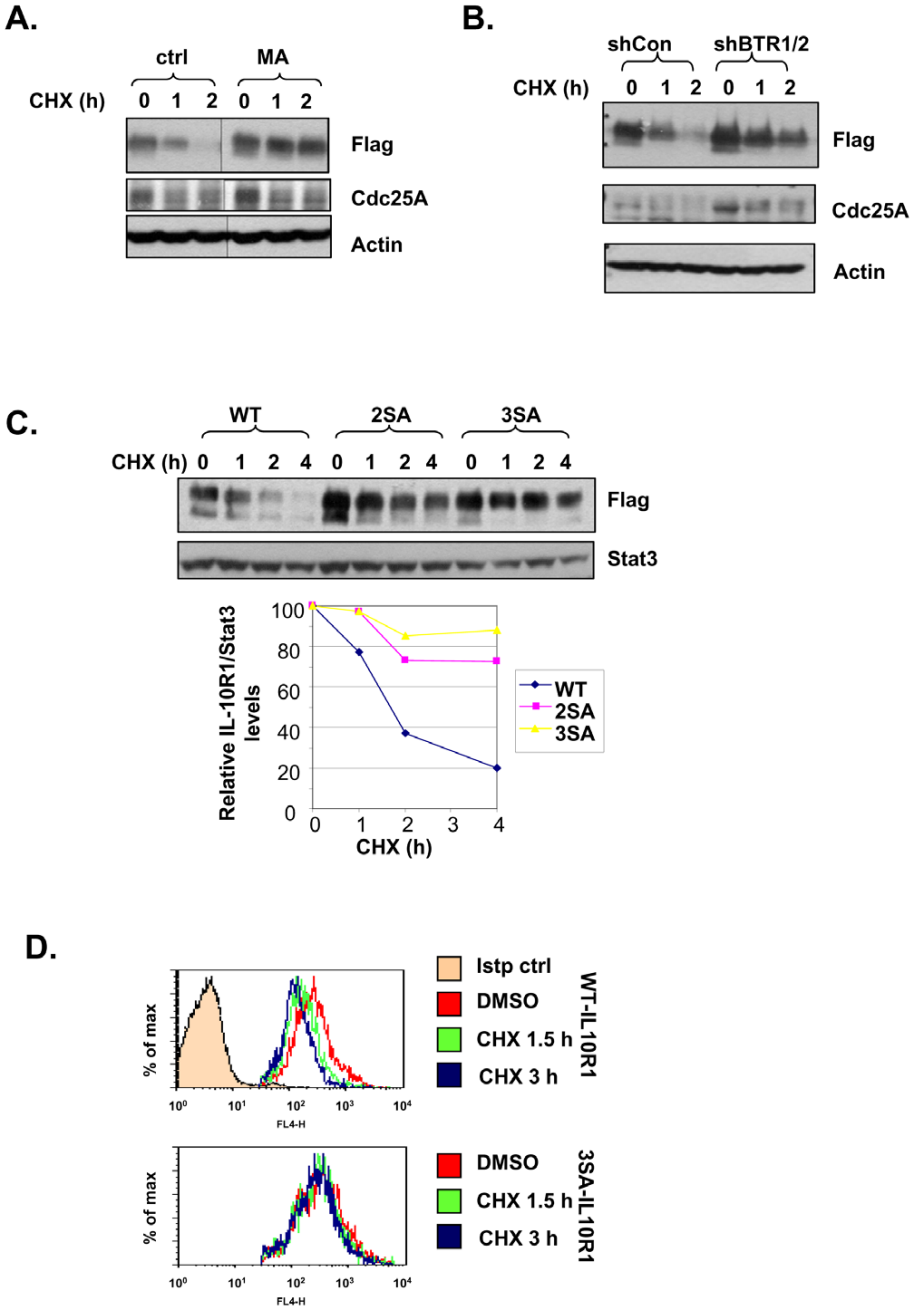
βTrCP-mediated ubiquitination drives IL-10R1 degradation. (A) 293T cells stably transfected with IL-10R1 were pretreated for 0.5 h with or without 20 mM of methylamine (MA); (B) 293T cells stably expressing IL-10R1 were transfected with shCon or shBTR1/2; (C) 293T cells stably transfected with the WT, S319, 23A (2SA) or S319, 23, 70A (3SA) IL-10R1 were used; (D) 293T cells stably expressing WT or 3SA IL-10R1 were used. In (A), (B), (C) and (D), all cells were treated with 25 µg/ml of cycloheximide (CHX) for indicated times. In (A), (B) and (C), the levels IL-10R1-Flag in each sample were determined by immunoblotting. The levels of Cdc25A were used as an indicator of βTrCP knockdown efficiency (B). In (C), band intensities were determined by NIH ImageJ. Levels of IL-10R1 relative to those of Stat3 (loading control) were calculated and graphed (WT: diamonds; 2SA: squares; 3SA: triangles). In (D), cell surface IL-10R1 levels were determined by FACS analysis. Only cells stained positive for IL-10R1 were gated.

Our data thus far indicate that the βTrCP-mediated ubiquitination of cell surface IL-10R1 leads to its down-regulation. We examined whether the latter mode of regulation is mediated by a ubiquitination-dependent enhancement of IL-10R1 endocytosis that promotes the receptor's endolysosomal degradation. To prevent the overloading of the endocytosis and vesicle trafficking systems by the transiently transfected IL-10R1, 293T cells stably expressing the WT or the mutant forms of IL-10R1 were used in experiments analyzing the receptor's post-ubiquitination fate (Fig. 4 and Fig. 5). The constitutive endocytic rates of WT IL-10R1 and its ubiquitination-deficient mutant (3SA-IL-10R1) were determined using an ELISA-based assay [49] that measures the kinetic disappearance of pre-labeled, cell surface IL-10R1 after allowance of its endocytosis (Fig. 4A). Within the time course of this experiment, the levels of internalized WT and 3SA IL-10R1 both increased over time. Nevertheless, 3SA IL-10R1 was endocytosed with a much slower rate compared to its WT counterpart (Fig. 4A). In addition, we adopted a confocal microscopy-based assay to assess IL-10R1 endocytosis [50]. The cell surface and intracellular IL-10R1 was stained sequentially with a green- and red-fluorescent secondary antibody, under stepwise non-permeant and permeant conditions. Using this visualizing method, the impaired constitutive endocytosis of 3SA IL-10R1 was further confirmed by the observation that the mutant-expressing cells consistently contain fewer and smaller red-fluorescent spots after being allowed to endocytose membrane proteins (Fig. 4B: lower two panels). These results strongly suggest that the βTrCP-mediated ubiquitination of cell surface IL-10R1 promotes its endocytosis.

Lysosomes are the principle cellular organelle responsible for degradation of the endocytosed membrane proteins [51]. To follow the turnover of IL-10R1, cells were treated with cycloheximide to inhibit protein synthesis. Consistent with the notion that IL-10R1 is degraded via the endolysosomal pathway, the constitutive decay of the total cellular IL-10R1 was effectively blocked by a lysosomal inhibitor, methylamine (Fig. 5A). Interestingly, a proteasomal inhibitor, MG132, also partially inhibited IL-10R1 degradation (data not shown), in agreement with a previous report [32]. However, since proteasome inhibition can lead to a myriad of secondary cellular events, including limiting the cellular pool of free ubiquitin [52], [53], we should be cautious not to over-interpret the latter data. We next proceeded to confirm the role of βTrCP-dependent ubiquitination of IL-10R1 in mediating its constitutive degradation. As expected, the shRNA-mediated knockdown of βTrCP1/2 notably stablized IL-10R1 (Fig. 5B). To corroborate these data, we also examined the basal turnover of the WT and the mutant forms of IL-10R1 that are partially (2SA) and largely (3SA) deficient in βTrCP binding (Fig. 2). As pool-to-pool variations commonly exist among stable transfectants, it was not surprising that the steady-state levels of various forms of IL-10R1 did not strictly follow an inverse relationship to their binding affinities for βTrCP (Fig. 5C: lane 1, 5 and 9). Nevertheless, the stability of the 3SA IL-10R1 was the greatest among the group, followed by that of the 2SA and the WT IL-10R1, sequentially. Taken together with the data from Fig. 5B, these results indicate that recruitment of βTrCP via the two DSG motifs of IL-10R1 jointly contributes to the receptor's constitutive degradation. It is important to note that neither βTrCP depletion nor mutation at the DSG motifs led to stabilization of the immature IL-10R1 (Fig. 5B, 5C), reiterating the selective regulation of mature IL-10R1 by βTrCP. Lastly, we directly measured the basal turnover of cell surface WT and 3SA IL-10R1 by FACS analysis. Cell surface levels of the 3SA IL-10R1 were less sensitive to the cycloheximide-induced down-regulation, as compared to those of the WT IL-10R1 (Fig. 5D). We conclude that the βTrCP-mediated ubiquitination of IL-10R1 promotes endocytosis and subsequent degradation of IL-10R1, whereby regulating the steady-state cell surface IL-10R1 levels.

**Figure 6:**
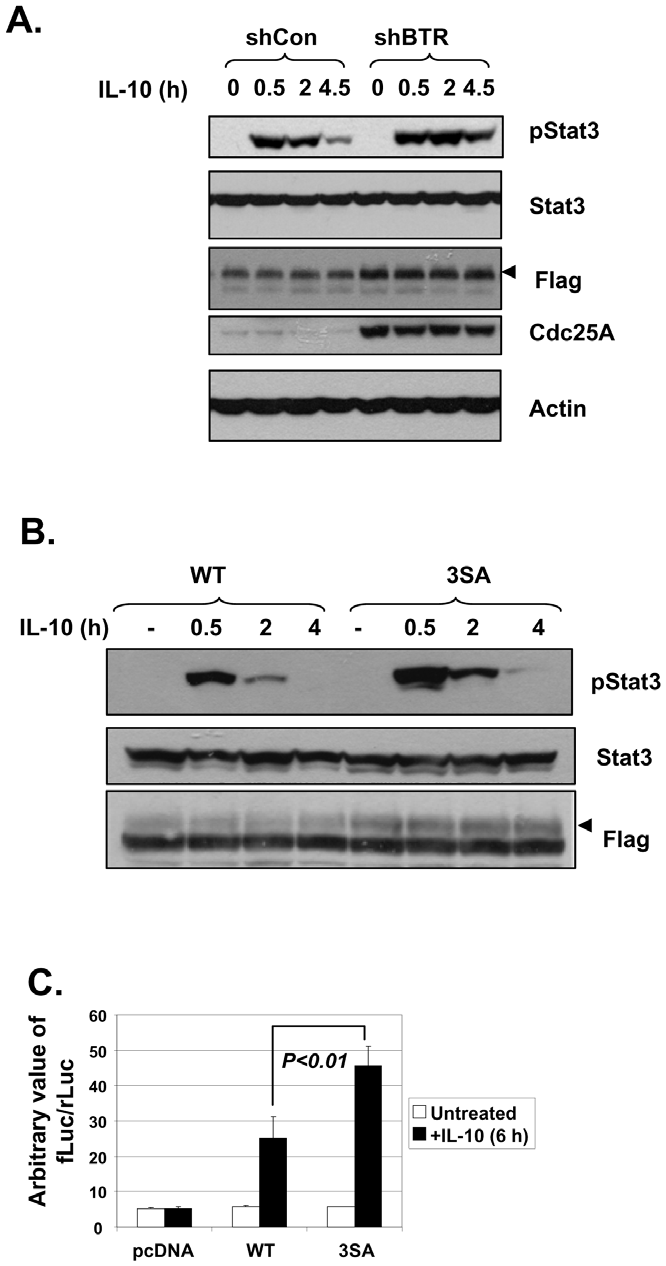
Inhibition of βTrCP-mediated IL-10R1 degradation leads to increased cellular responsiveness to IL-10. (A and B): 293T cells stably expressing IL-10R1 were transfected with shCon or shBTR1/2 (A); 293T cells were transiently transfected with WT or 3SA IL-10R1 (B). Cells were treated with 5 ng/ml of IL-10 for indicated times and the levels of pStat3, Stat3, IL-10R1-Flag were determined by IB. The bands representing the mature form of IL-10R1 were denoted by arrow heads in the Flag panels. In (A), the levels of another βTrCP substrate, Cdc25A, were used to show the effectiveness of βTrCP knockdown. (C) Raw264.7 cells were transfected with pcDNA, WT or Ser320, 24, 67A (3SA) mIL-10R1, together with the M67-luciferase reporter construct and the pCMV-renilla luciferase control. Cells were treated with IL-10 (black bars) or without (open bars) for 6 h and samples were subjected to dual-luciferase activity assay. Cells were transfected in triplicates. Average Stat3 reporter activities represented by the relative ratios of firefly over renilla luciferase activities (fLuc/rLuc) were shown on the graph, with error bars showing standard deviations. Comparison was made between IL-10-treated WT versus 3SA groups and the *p* value was shown. Data shown are a representative from two independent experiments.

**Figure 7:**
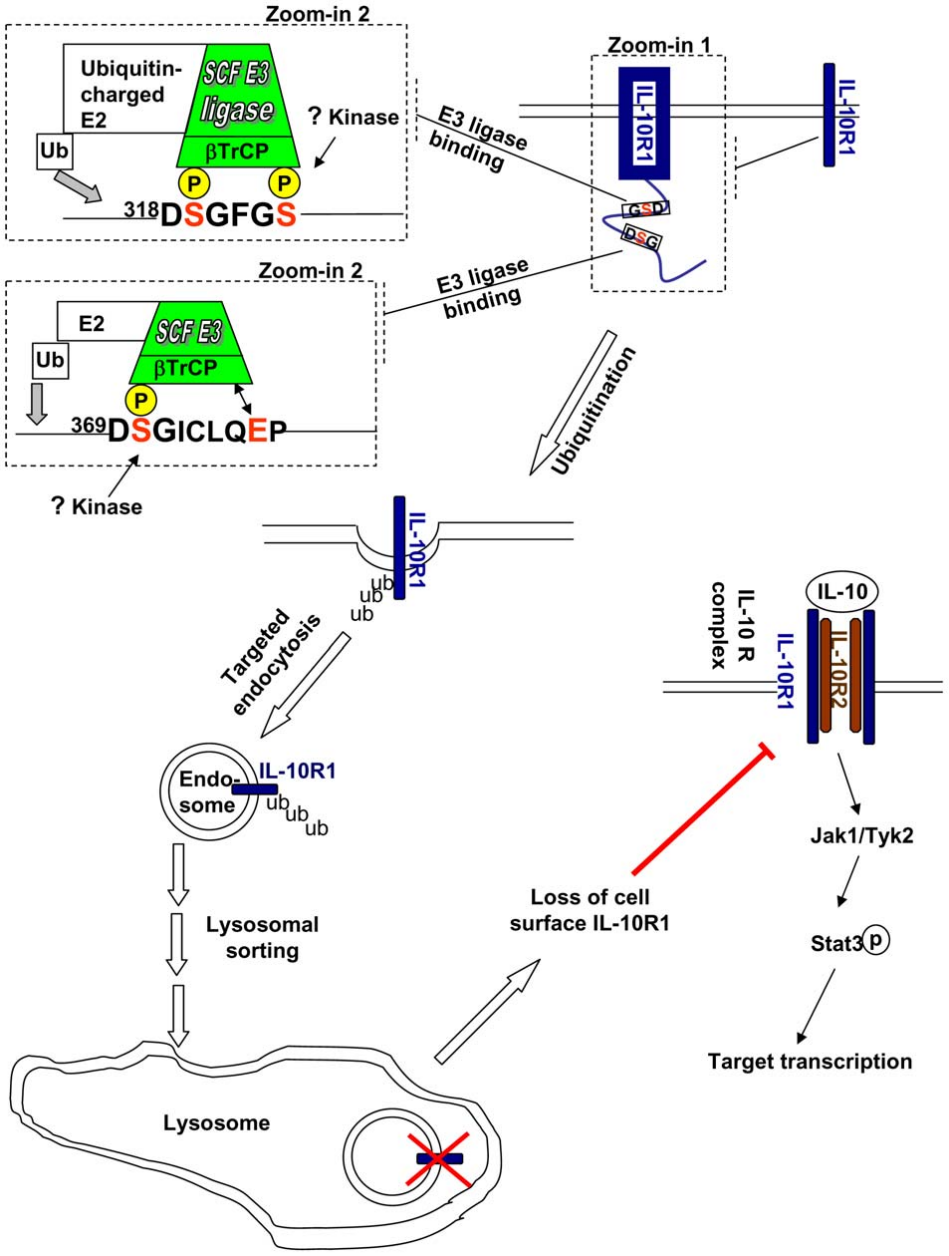
The major findings of the present study are summarized in a model. IL-10R1 contains two βTrCP-binding sites (^318^DSGFGS and ^368^DSGICLQEP) in its cytoplasmic tail (see the dash-lined box marked by ‘Zoom-in 1’). The phosphorylation of the serine residues within these two sites can additively trigger recruitment of SCF^βTrCP^ ubiquitin E3 ligase, resulting in ubiquitination of cell surface IL-10R1 (see the dash-lined boxes marked by ‘Zoom-in 2’). The ubiquitination of IL-10R1 promotes its endocytosis that expedites the latter’s eventual degradation via the lysosome. The nature of the physiologically relevant stimuli and of the associated kinase(s) that trigger phophorylation of the two βTrCP-binding sites within IL-10R1 is not known at this point. Nevertheless, in ectopically transfected IL-10R1, these sites appear to be both engaged, which results in the negative regulation of cell surface IL-10R1 levels. Potentially, under certain physiological/pathological conditions that trigger DSG motifs phosphorylation, the βTrCP-mediated regulation of cell surface IL-10R1 may play an important role in determining the magnitude of IL-10-mediated Jak/Stat signaling and transcriptional regulation.

As the levels of IL-10R1 correlate with the cellular responsiveness to IL-10 [30], we hypothesized that the βTrCP-dependent modulation of IL-10R1 degradation and abundance can influence IL-10 function in its target cells. Indeed, compared to that in the control cells, phosphor-Stat3 (pStat3) induction by IL-10 is more substantial in cells that had βTrCP knocked down (Fig. 6A). Similar results were obtained when the levels of IL-10-induced pStat3 were compared in cells expressing the WT versus the βTrCP binding-deficient, 3SA IL-10R1 (Fig. 6B). Such enhancement of IL-10 signaling in these two different models harboring defects of the βTrCP-IL-10R1 degradation axis is apparently correlated with the higher levels of mature IL-10R1 therein (the arrow heads in Flag panels, Fig. 6A, 6B). Lastly, we examined whether the βTrCP-IL-10R1 negative regulatory pathway exists in immune-related cells. We utilized a mouse macrophage/monocyte cell line, Raw264.7. These cells have been shown to express a functional IL-10 receptor [54]. However, we noticed that IL-10 signaling initiated by the endogenous IL-10 receptor was not sufficient to trigger a marked increase in the activity of a transfected Stat3-luciferase reporter (Fig. 6C, ‘pcDNA’). Co-transfection of the WT IL-10R1 with the reporter resulted in the latter's sensitivity to IL-10 treatment. Importantly, expression of the 3SA mutant IL-10R1 poised the cells to exhibit a more robust induction of Stat3-reporter activity in response to IL-10 (Fig. 6C). In summary, we have identified a βTrCP-dependent pathway that can negatively regulate IL-10R1 levels and subsequently, the magnitude of the cellular responses to IL-10 (Fig. 7). Given the conservation of the apparent βTrCP recognition motifs within IL-10R1 from different species, the βTrCP-IL-10R1 axis identified in the current study may represent an important regulatory pathway modulating IL-10 function *in vivo*.

## Discussion

We show in the present study that βTrCP can bind to a ^318^DpSGFGpS and a ^369^DpSGICLQE motif of IL-10R1 in a phosphorylation-dependent manner (Figs. 1 and 2), leading to its ubiquitination (Fig. 3), endocytosis (Fig. 4) and lysosomal degradation (Fig. 5). Consistently, inhibition of this pathway in an IL-10R1 ectopic expression system leads to the receptor's stabilization (Fig. 5) and up-regulation (Fig. 2B, 2C; Fig 3C, 3D and Fig. 6A, 6B), which improves the cellular responsiveness to IL-10 (Fig. 6). Therefore, our data suggest that IL-10R1 may represent a novel substrate of βTrCP. Nevertheless, further investigation into the natures of the potential stimuli that may induce βTrCP-mediated regulation of IL-10R1 is critically needed to confirm the biological relevance of our findings. Several possibilities exist. Firstly, although we did not observe IL-10-dependent recruitment of βTrCP to IL-10R1 in our experimental system (Fig. 1C), it is formally possible that such an event may be sufficiently triggered by IL-10 signaling under certain cellular context. The fact that a previously identified domain (aa282 to aa389) responsible for IL-10-dependent IL-10R1 down-regulation in a pro-B cell line encompasses both βTrCP recognition elements is in agreement with such a notion[32]. Secondly, recent studies in the IFNAR1 system have indicated that phosphorylation of a βTrCP-binding site within a cytokine receptor can indeed be robustly induced by certain ligand-independent stimuli [34], [37], [38], [39]. It is rather conceivable that a similar scenario may apply to IL-10R1, which commits the receptor to degradation in response to selected physiological or pathological signals. In this regard, one shall be intrigued by the fact that IL-10R1 indeed possesses two βTrCP-binding motifs that are conserved in mammals. More than one βTrCP-binding motif has been previously identified in Gli/Ci [55], [56]. However, unlike the latter sites in Gli/Ci that are phosphorylated by similar upstream kinases, the serine residues within the two βTrCP-binding sites of IL-10R1 are surrounded by sequences that show little similarities between one and the other. We therefore hypothesize that the phosphorylation of these two DSG motifs is controlled by distinct kinases, providing the flexibility of regulating receptor degradation under different conditions. The investigations testing these hypotheses are currently underway.

Regulation of membrane receptors' endocytosis and intracellular sorting by targeted ubiquitination has emerged as a major regulatory mechanism that determines their abundance and signaling [57], [58]. In regard to ubiquitin-mediated induction of endocytosis, modification of acceptor lysine residues by either mono-ubiquition or poly-ubiquitination can be sufficient, dependent on the receptor of the interest and the associated cellular context [59]. Moreover, it has been established that ubiquitin moieties conjugated to a given membrane substrate may sufficiently serve as direct endocytic signals [57], or they may cooperate with other pre-existing endocytic motifs on the target molecules to promote endocytosis [60]. It will be of great interest to identify the acceptor lysine residues on IL-10R1 and the topology of the conjugated ubiquitin chains, as well as to elucidate their roles in regulating IL-10R1 endocytosis. In addition to regulation of endocytosis, ubiquitination of a membrane receptor may also play an important role in its post-endocytic sorting into the lysosomes [58]. Whether such a paradigm also applies to the IL-10R1 system awaits formal elucidation. Following this line of discussion, it is interesting to point out that the topology of ubiquitin chains driving the lysosomal sorting can divert from that triggering the upstream endocytosis event [60], [61]. Therefore, potential ubiquitin chain-editing mechanisms may also contribute to regulating the cellular destination of the endocytosed IL-10R1 [51]. To summarize, we believe that our study has provided a basis for follow-up investigations into the detailed mechanisms regulating distinct stages of IL-10R1 endocytic trafficking. Such future works may also improve our knowledge of the fundamental mechanisms that regulate cell surface receptor trafficking in general.

Our findings add another member of the cytokine receptor family to the list of βTrCP substrates that has already included IFNAR1, PRLR, Epo-R and GHR [33], [44], [45], [62]. Cytokine receptors can be divided into class I and class II, based mainly on the relative positioning of the four conserved cysteine residues in the extracellular fibronectin III repeats, together with several other structural determinants (reviewed in [63]). Of the cytokine receptors regulated by βTrCP, IL-10R1 and IFNAR1 belong to the class II cytokine receptor family that currently includes 9 other members in mammals. Class II cytokine receptors appeared before invertebrate and vertebrate diversion and expanded rapidly in early vertebrates. One apparent class II cytokine receptor can be identified in sea squirt *Ciona intestinalis* (GI: 198428337)[63] and in amphioxus *Branchiostoma floridae* (GI: 260796075 [64]), whereas zebrafish *Danio rerio* and pufferfish *Tetraodon nigroviridis* genomes encode similar numbers of the class II cytokine receptors compared to those in the mammalian genomes [63], [65]. Importantly, we have clearly identified putative βTrCP sites in fish orthologs of IFNAR1 (CRFB5) and IL-10R1 (CRFB7), at positions similar to those within their counterparts in higher organisms (Figure S4 and Figure S5). Interestingly, no apparent DSG motifs can be identified within the putative ancestor form of class II cytokine receptor from *C. intestinalis* or *B. floridae*, or within the other 9 members of the mammalian class II cytokine receptors. This suggests that the β-TrCP-dependent regulation of IFNAR1 or IL-10R1 was acquired during their own diversification from ancestors under the pressure of evolving a sophisticated immune system [63], [65], and such a mode of regulation is likely crucial for the proper regulation of these receptors during immune responses elicited by pathogens. It is important to note that from flies to human, the activation of NF-κB (Dorsal), an essential node of host defense program, is also under the control of βTrCP (Slimb) that targets IκB (Cactus) for ubiquitination and degradation [66]. Since changes in βTrCP levels/activities may coordinately affect the degradation rate of its multiple substrates [67], it is thus conceivable that the acquisition of βTrCP-mediated regulation of IFNAR1 and IL-10R1 in vertebrates would lead to an additional level of cross-talks among the type I IFN-induced signaling, the IL-10-dependent signaling and the βTrCP-IκB-NF-κB axis. This may hypothetically favor the development of a more balanced immune response against pathogens. Although such a hypothesis is highly speculative, the apparently selective clustering of βTrCP substrates in several categories of cellular processes (e.g., mitosis, growth regulation, immune regulation, transcriptional regulation, circadian clock regulation) implies that the substrate repertoire of βTrCP is not determined randomly during evolution. Nevertheless, it is obvious that βTrCP binding to IFNAR1 or IκB is regulated by activation of different upstream kinases attributed to non-overlapping environmental stimuli [36], [68], [69]. Likewise, it should be at least conceivable that phosphorylation of the DSG motifs of IL-10R1 is regulated by upstream signals that are relatively independent from those affecting IFNAR1 or IκB. Taken together, we suggest that multi-layers of selective pressure have shaped the modes of βTrCP-mediated regulation of IFNAR1 and IL-10R1, which likely contributes to the increased complexity of immune regulation from invertebrates to vertebrates.

Despite seemly representing an evolutionarily conserved mechanism, there exists the possibility that the βTrCP-IL-10R1 axis uncovered in the present study may become dys-regulated under certain conditions of immune imbalance, analogous to what has been shown in the IFNAR1 system during infections by some viruses [34], [38]. Indeed, decreased IL-10 receptor levels have been observed in cells derived from rheumatoid arthritis joints [18] or in cells exposed to a microbial cell wall component, zymosan [19], although the underlying mechanisms will await further elucidation. Owing to the importance of IL-10 and its receptor in maintaining the immune homeostasis, we speculate that alterations in βTrCP-IL-10R1 axis may represent an intriguing possibility involved in the pathogenesis of certain inflammatory conditions.

## Materials and Methods

### Plasmids and Reagents

C-terminally Flag-tagged IL-10R1 plasmid used throughout in the present study was generated via sub-cloning of hIL-10R1 coding sequence from pIRESpuro3-IL-10R1 (a kind gift from Dr. C Gasche, University of Vienna, Austria [70]) into the pcDNA vector. The Flag-tagged mIL-10R1 was prepared from MIGR1-mIL-10R1 vector (a generous gift from Dr. D Yu, University of Pennsylvania), and also sub-cloned into pcDNA. Mutagenesis was performed using Takara's Prime Star DNA polymerase according to the manufacture instructions. Human βTrCP2 expression construct in backbone vector pEF was a kind gift from Dr. SY Fuchs (University of Pennsylvania). The HA-ubiquitin expression plasmid was generously provided by Dr. Y Yarden (Weizmann Institute of Science, Israel). M67-luciferase construct was provided by Dr. JF Bromberg (Memorial Sloan-Kettering Cancer Center). Renilla luciferase control construct was from Promega. shRNA vector (pLKO.1) and shRNA control (shC002) was purchased from Sigma. Hairpin sequence targeting both human βTrCP1 and 2 ([47], GTGGAATTTGTGGAACATC) was cloned into pLKO.1. Recombinant human IL-10 was purchased from R&D systems. Cycloheximide was from Sigma. λ-phosphatase was purchased from Santa Cruz. Dual luciferase assay kit was purchased from Promega.

Cell culture, transfections: 293T cells and Raw264.7 cells were purchased from ATCC and were maintained in Dulbecco's modified Eagle's medium (DMEM) supplemented with 10% (v/v) fetal bovine serum (Hyclone) unless otherwise specified. 293T cells and Raw264.7 cells were transfected with Lipofectamine 2000 reagent (Invitrogen) and Nano Juice reagent (EMD Chemicals) respectively. 293T stably expressing IL-10R1 was prepared by transfecting cells with pcDNA-IL-10R1 together with pLKO.1 (at a 50:1 ratio), followed by selection with puromycin (1 µg/ml for 3 weeks). Puromycin-resistant clones were pooled and expanded. Such transfection and selection scheme yields about 25 to 30 percent cells stably expressing IL-10R1.

Immunotechniques: Antibodies against pStat3, Stat3 (Cell Signaling), against IL-10R1 (polyclonal, C20 or N20), hIL-10R1 (monoclonal, 3F9), Cdc25A, ubiquitin (P4D1), HA tag (Y-11 or 12CA5) from Santa Cruz and against Flag tag and β-actin (Sigma) were used for immunoprecipitation and immunoblotting. Goat anti-rabbit and goat anti-mouse secondary antibodies were purchased from Thermo Scientific. Polyclonal rabbit antibody against pSer319, 23 of hIL-10R1 (pSer320, 4 of mIL-10R1) was generated by Signalway Antibody (TX, USA). When not indicated specifically, cells lysis was performed in buffer containing 1% Triton-X 100, 0.5% NP-40, 150 mM NaCl, 10 mM Tris-HCl pH 7.5, 1 mM EDTA, 1 mM NaF, 1 mM sodium orthovanadate, protease inhibitor cocktail (sigma, 1∶500 dilution) and freshly added 1 mM PMSF. In some experiments, cells were lysed in RIPA buffer (25 mM Tris-HCl pH 7.6, 150 mM NaCl, 1% NP-40, 1% sodium deoxycholate, 0.1% SDS with further addition of EDTA, NaF, sodium orthovanadate, protease inhibitor cocktail and PMSF, as described above). Immunoprecipitation (IP) was carried out using indicated antibodies in combination with protein G agarose (Invitrogen).

For *in vitro* βTrCP binding assay, the IL-10R1 IPed from the RIPA lysate was first washed thoroughly with the Triton X-100-based lysis buffer. λ-phosphatase treatment of the washed beads was carried out at 30 degrees for 30 min, in 20 µl of phosphatase buffer (50 mM HEPES, pH 7.5, 100 µM EGTA, 5 mM dithiothreitol, 0.1% BRIJ 35, with freshly added 2 mM MnCl_2_) containing 200 units of the enzyme. The reaction was stopped by placing the samples on ice and the beads were washed with the Triton X-100 lysis buffer. 50 µl of the lysates from 293T cells transfected with HA-βTrCP2 was added to the beads and the slurry was incubated further at 4 degrees for 1 h with constant agitation. At the end of the incubation, the unbound material was removed from the beads by washes.

Cell surface immunoprecipitation [71] was performed to enrich the cell surface IL-10R1 for analysis. Briefly, cells were dissociated from the plates with PBS containing 1 mM EDTA. After incubated with 5 µg/ml of the antibody specific to the extracellular domain of IL-10R1 (3F9) on ice, the cell suspension was washed extensively with PBS. The cell pellets were lysed using the Triton X-based lysis buffer. Protein G beads were added to the lysates to pull down the cell surface IL-10R1. In some experiments, the remaining supernatants were subjected to a second round of immuno-precipitation to subsequently enrich intracellular IL-10R1. To examine the levels of direct ubiquitin modification of IL-10R1, the natively IPed materials were subjected to denaturing Flag IP (see below).

All protein samples were run on SDS-PAGE gels and were ‘wet’-transferred to PVDF membranes in standard Tris-glycine buffer. Immuno-blotting analysis was carried out using antibodies at manufacture suggested dilutions, and the immuno-reactivity was determined by using chemiluminescent substrate (Millipore).

### Denaturing immunoprecipitation

To examine the levels of IL-10R1 ubiquitination, IL-10R1-containing protein complexes were first IPed under native conditions. The IPed materials were boiled in 20 µl of denaturing lysis buffer (50 mM Tris-HCl pH 7.6, 150 mM NaCl and 2% SDS) for 10 min to break the protein complexes. The samples were diluted 1:20 using the Triton X-100 lysis buffer (as described above) and the Flag antibody/protein G beads were added for a second time to purify the denatured Flag-IL-10R1. When HA-ubiquitin was co-transfected with the IL-10R1-Flag, a similar denaturing IP using a monoclonal antibody against HA was used.

### Endocytosis assay

A cell surface ELISA-based endocytosis assay [49] was adopted and slightly modified to measure the ligand-independent endocytotic rates of IL-10R1. Briefly, 293T stable transfectants were plated on poly-D-lysine-coated 24-well plates until reaching confluency. All subsequent steps were performed on ice and with ice-cold reagents, unless otherwise specified. Cells were washed in PBS containing 0.66 mM of CaCl_2_ and 0.33 mM of MgCl_2_ (PBS++) and incubated in PBS++ buffer added with 0.5% BSA (PBS++BSA) for 20 min. Cells were then incubated with 5 µg/ml of antibody specific to the extracellular domain of IL-10R1 (3F9, rat IgG2a, Santa Cruz) in the PBS++BSA for 45 min. After antibody removal and washes, cells were added with pre-warmed culture medium and the plates were placed into the 37-degree incubator to allow IL-10R1 endocytosis. After the specified periods of time, cells were then placed back on ice. After two washes with PBS++, the cells were incubated with 67 ng/ml of HRP-conjugated secondary antibody (Santa Cruz) in the PBS++BSA for 30 min. After three washes, cells were added with 0.1 mg/ml of HRP substrate tetramethylbenzimdine (TMB, Sigma) in the substrate solution (0.2 M sodium citrate, pH 4.0, 0.03% H_2_O_2_) and incubated for 15 min. Equal volume of 2N HCl was then added to each well and the color development was measured using a plate reader (SH-1000, Hitachi) under 450 nm. Cell-associated HRP activity represents the levels of antibody-labeled IL-10R1 remaining on the cell surface. The values were calibrated according to the levels of crystal violet staining to control the variations of cell numbers. The levels of endocytosis were calculated by the amount of signal loss at each time point relative to the baseline signal from time 0. Two wells of cells were processed individually for each experimental condition. The background signal from samples stained with the control IgG was subtracted from every sample. The average values and the range of data from the duplicated samples were graphed.

### Confocal microscopy

To visualize the endocytosed IL-10R1, a method described in [50] was adopted. Cell surface IL-10R1 in 293T stable transfectants was first pre-labeled with antibody as stated in the above section. Cells were placed back into the 37-degree incubator to allow IL-10R1 endocytosis for 45 min. Cells at 0 and 45 min were fixed on ice with 0.25% PFA in PBS for 1 h. The fixed cells were then incubated with an Alexa488-conjugated donkey anti-rat secondary antibody (green, Invitrogen) for 2 h on ice to stain the remaining cell surface IL-10R1. After washes, cells were then quickly permeablized with cold methanol to make the endocytosed IL-10R1 accessible. A second Cy3-conjugated donkey anti-rat antibody (red, Jackson ImmunoResearch Laboratories) was subsequently added to the samples to stain the endocytosed IL-10R1. The coverslips were mounted using an anti-fade reagent containing DAPI (P36935, Invitrogen). The pictures were taken under an Olympus FV-1000 confocal microscope and representative three-color-overlaid pictures were presented.

### Flow cytometry

Cells surface levels of transfected IL-10R1 were determined by staining cells (dissociated using 1x PBS containing 2 mM EDTA) with anti-IL10-R1 (3F9), in combination with anti-rat-biotin (Biolegend) and streptoavidin-APC (Biolegend). Cell surface antigen levels were examined by FACSCaliber flow cytometer (BD Pharmingen). The data were analyzed by the FlowJo program (Tree Star).

### Luciferase assay

Raw264.7 cells in 24 wells were transfected with pcDNA (0.075 µg), CMV-rLuc (0.05 µg) and M67-fLuc (0.125 µg) plasmids using Nanojuice transfection reagent (EMD Chemicals). On the next day, cells were treated for 6 h with IL-10 (10 ng/ml) and harvested using the lysis buffer provided in the Dual-luciferase assay kit (Promega). Samples were mixed with the Dual-luciferase substrate in 4 ml round-bottom polystyrene tubes (BD Biosciences) and the levels of luminescence were determined using Lumat LB 9507 (Berthold Technologies, Germany).

## Supporting Information

Figure S1 **Phosphorylation of Ser320,4 in transfected mouse IL-10R1.**
(PDF)

Figure S2 **Interaction of mouse IL-10R1 with βTrCP is mediated by Ser320, Ser324 and Ser367.**
(PDF)

Figure S3 **Western blot analyses using two different antibodies reveal that βTrCP inhibits the expression of mature IL-10R1.**
(PDF)

Figure S4 **Two DSG motifs similar to those found in human IL-10R1 can be identified in the zebrafish ortholog of IL-10R1.**
(PDF)

Figure S5 **A conserved DSGXYS motif is present in the zebrafish ortholog of IFNAR1.**
(PDF)
