# Supplementary material for: Regulation of Interleukin-10 Receptor Ubiquitination and Stability by Beta-TrCP-Containing Ubiquitin E3 Ligase
Source: PLoS One. 2011 Nov 8;6(11):e27464. doi: 10.1371/journal.pone.0027464 (PMC3210801; doi:10.1371/journal.pone.0027464)
Supplement: Figure S1 — Phosphorylation of Ser320,4 in transfected mouse IL-10R1. (PDF) [file pone.0027464.s001.pdf]

Figure S1

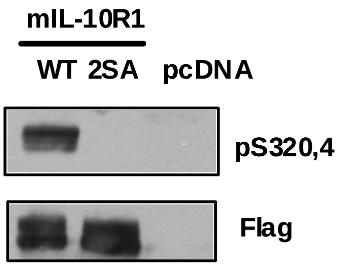

Figure S1: Phosphorylation of Ser320,4 in transfected mouse IL-10R1. 293T cells were transfected with the WT or the S320, 4A (2SA) murine IL-10R1. Cell lysates were subjected to IB with pSer320, 4-specific antibody and Flag antibody.
