# Supplementary material for: Regulation of Interleukin-10 Receptor Ubiquitination and Stability by Beta-TrCP-Containing Ubiquitin E3 Ligase
Source: PLoS One. 2011 Nov 8;6(11):e27464. doi: 10.1371/journal.pone.0027464 (PMC3210801; doi:10.1371/journal.pone.0027464)
Supplement: Figure S2 — Interaction of mouse IL-10R1 with βTrCP is mediated by Ser320, Ser324 and Ser367. (PDF) [file pone.0027464.s002.pdf]

Figure S2

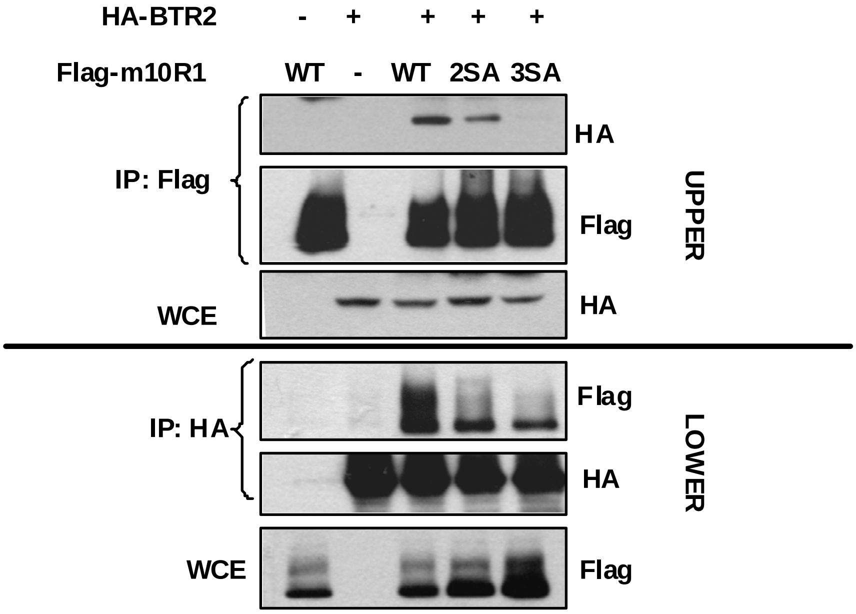

Figure S2: Interaction of mouse IL-10R1 with βTrCP is mediated by Ser320, Ser324 and Ser367. 293T cells were co-transfected with WT, S320,4A (2SA) or S320,24,67A (3SA) murine IL-10R1 and HA-βTrcp2 (HA-BTR2). In the upper panels, lysates were IPed with Flag antibody and the immuno-precipitates were subjected to IB for associated HA-BTR2 and total levels of IL-10R1-Flag in each IP. Whole cell extract (WCE) samples were IBed for the total levels of transfected HA-BTR2. In the lower panels, lysates were IPed with HA antibody and the immunoprecipitates were subjected to IB for associated IL-10R1-Flag and the total levels of HA-BTR2. WCE samples were IBed for the total levels of recombinant IL-10R1.
