# Supplementary material for: Regulation of Interleukin-10 Receptor Ubiquitination and Stability by Beta-TrCP-Containing Ubiquitin E3 Ligase
Source: PLoS One. 2011 Nov 8;6(11):e27464. doi: 10.1371/journal.pone.0027464 (PMC3210801; doi:10.1371/journal.pone.0027464)
Supplement: Figure S3 — Western blot analyses using two different antibodies reveal that βTrCP inhibits the expression of mature IL-10R1. (PDF) [file pone.0027464.s003.pdf]

Figure S3

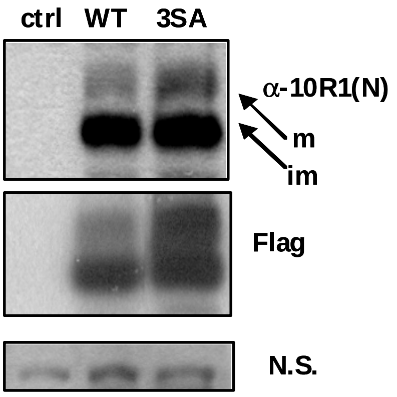

Figure S3: Western blot analyses using two different antibodies reveal that βTrCP inhibits the expression of mature IL-10R1. 293T cells were transfected with WT or Ser319, 23, 70A (3SA) hIL-10R1. Lysates were examined by IB using an antibody specifically recognizing the N'-terminus of IL-10R1 (α-10R1 (N)) or Flag. The mature and immature forms of IL-10R1 are marked by arrows in the α-10R1 (N) panel. A non-specific (N.S.) band is used as loading control.
