# Supplementary material for: Regulation of Interleukin-10 Receptor Ubiquitination and Stability by Beta-TrCP-Containing Ubiquitin E3 Ligase
Source: PLoS One. 2011 Nov 8;6(11):e27464. doi: 10.1371/journal.pone.0027464 (PMC3210801; doi:10.1371/journal.pone.0027464)
Supplement: Figure S4 — Two DSG motifs similar to those found in human IL-10R1 can be identified in the zebrafish ortholog of IL-10R1. (PDF) [file pone.0027464.s004.pdf]

Figure S4

```
ct-h10R1     V-IIFFAFVLLLSGALAYCLALQLYVRRRKKLPSVLLFKKPSPFIFISQRPSPETQDTIH
ct-zCRFB7    IGVIGVVGVITL-----FMLFVCFFLRRPGKMPAVLKLAVNGWL------PMNVGQTEVE
             : :* .. *: *     : * : :::** *:*:** :   . :      *    *  :.

ct-h10R1     PLDEEAFL----KVSPELKNLD-----------LHGSTDSGFG-----STKPSLQTEEPQ
ct-zCRFB7    SVTQKGWLLNSNKIAEKTKAFDEIEELSEDEKERRESTDSGVSIGQQDSIKNRPQREEDS
             .: ::.:*    *:: : * :*             : *****..     * *   * ** .

ct-h10R1     FLLPDPHPQADRTLGNR---EPPVL--GDSCSSGSSNSTDSGICLQEPSLSPSTGPTWEQ
ct-zCRFB7    GCGSLTGTEDSLSSGRRSLEELPFLDGGGNSSSVEGTREDSGLGIQTQDISDKPKPM---
                . . .: . : *.*   * *.*  *...** ...  ***: :*  .:* .. *

ct-h10R1     QVGSNSRGQDDSGIDLVQNSEGRAGDTQGGSALGHHSPPEPEVPGEEDPAAVAFQGYLR-
ct-zCRFB7    --------HDDLLSEIVVIGDGYRSQSPSAEA-----ETETTIQCDEDANMVSRTNGYRS
                     :**   ::*  .:*  .:: ...*      .*.  :  :**.  *:  .   *

ct-h10R1     -QTRCAEEKATKTGCLEEESPLTDGLGPKFGRCLVDEAGLHPPALAKGYLKQDPLEMTLA
ct-zCRFB7    GQVTCLCSDSETCMWCKTRKHLTDCDSFSHKQTVNDND-------RSSYLKKSPLETVNM
              *.  *  ..: .     : ..  ***  . ..  :  : *:         ..***:.*** .

ct-h10R1     SSGAPTGQWNQPTEEWSLLALSSCSDLGISDWSFAHDLAPLGCVAAPGGLLGSFNSDLVT
ct-zCRFB7    FGLDDLSCHSDKTEESSLF---------------------ITCPLLLKEPYKLD--T
              .     .   .: *** **:                        ::.*  *  .:: *  *

ct-h10R1     LPLISSLQSSE----
ct-zCRFB7    LPL--TLGDVELTFT
             ***  :* . *
```

Figure S4: Two DSG motifs similar to those found in human IL-10R1 can be identified in the zebrafish ortholog of IL-10R1. Alignment of the cytoplasmic tail of hIL-10R1 (ct-h10R1) and zebrafish CRFB7 (ct-zCRFB7) is done using the online MAFFT program [1]. The alignment is shown in ClustalW format. The '*'s denote identical amino acid residues; ':'s denote conserved amino acid substitutions and '.'s denote semi-conserved amino acid substitutions. The two conserved DSG motifs were highlighted in yellow and blue, respectively.

1. Katoh, K., K. Misawa, K. Kuma, and T. Miyata. 2002. MAFFT: a novel method for rapid multiple sequence alignment based on fast Fourier transform. Nucleic Acids Research 30: 3059-3066.
