# Supplementary material for: Regulation of Interleukin-10 Receptor Ubiquitination and Stability by Beta-TrCP-Containing Ubiquitin E3 Ligase
Source: PLoS One. 2011 Nov 8;6(11):e27464. doi: 10.1371/journal.pone.0027464 (PMC3210801; doi:10.1371/journal.pone.0027464)
Supplement: Figure S5 — A conserved DSGXYS motif is present in the zebrafish ortholog of IFNAR1. (PDF) [file pone.0027464.s005.pdf]

Figure S5

```
ct-hIFNAR1      IVGICIALFALPFVIYAAKVFLRCINYVFFPSLKPSSSIDE-YFSEQPLKNLLLSTSEEQ
ct-zCRFB5       LALVLVFLLVLLLFLCSYRTF-QTFKSVCRPSAQLPAHIQELWLSDADTPQILL--SKEC
                :. : : *:.* :.: : :.* : :: *  ** : .: *:* ::*:    ::**  *:*

ct-hIFNAR1      IEKCFIIENISTIATVEETNQTDEDHKKYSSQTSQDSGNYSNEDESESKTSEELQ-----
ct-zCRFB5       VCEHMDVALVCAVDTHIPEEPQDSGGCSSDTHSSGDSGVYSEEDSATHTHSHSLSLKNTH
                : : : :  :.:: *    :  *..  . .:::* *** **:**.:  . *..*.

ct-hIFNAR1      --------QDFV
ct-zCRFB5       THDHTLLLTDTL
                         * :
```

Figure S5: A conserved DSGXYS motif is present in the zebrafish ortholog of IFNAR1. Alignment of the cytoplasmic tail of hIFNAR1 (ct-hIFNAR1) and the zebrafish CRFB5 (ct-zCRFB5) is done as in Fig. S4. The conserved βTrcp site is highlighted in yellow.
